# Circulating osteopontin and its association with liver fat content in non-obese women with polycystic ovary syndrome: a case control study

**DOI:** 10.1186/s12958-018-0331-4

**Published:** 2018-03-27

**Authors:** Yuying Wang, Wei Zhou, Chunhua Wu, Yi Zhang, Tzuchun Lin, Yun Sun, Wei Liu, Tao Tao

**Affiliations:** 1 Department of Endocrinology and Metabolism, Renji Hospital, School of Medicine, Shanghai Jiaotong University, 160 Pujian Road, Shanghai, 200127 China; 2 Department of Emergency, South Campus, Renji Hospital, School of Medicine, Shanghai Jiaotong University, Shanghai, China; 3 Division of Ultrasonography, Department of Radiology, Renji Hospital, School of Medicine, Shanghai Jiaotong University, 160 Pujian Road, Shanghai, 200127 China; 4 Shanghai Key laboratory for Assisted Reproduction and Reproductive Genetics, Center for Reproductive Medicine, Renji Hospital, School of Medicine, Shanghai Jiaotong University, 160 Pujian Road, Shanghai, 200127 China

**Keywords:** Polycystic ovary syndrome, Osteopontin, Liver fat content, Hyperandrogenism

## Abstract

**Background:**

Osteopontin (OPN) plays an important role in inflammatory processes and insulin resistance. Polycystic ovary syndrome (PCOS) is a reproductive metabolic disease associated with insulin resistance and metabolic abnormalities, including high levels of liver fat content (LFC). The objective of this study was to explore whether circulating OPN independently contributes to elevated LFC in non-obese PCOS patients.

**Methods:**

This study included 61 non-obese PCOS patients and 56 age-matched healthy women from Shanghai, China. After an overnight fast, all participants underwent anthropometric measurements, oral glucose tolerance tests, lipid profile and sex hormone measurements. Quantitative measurement of LFC by ultrasonography was performed. OPN concentrations were measured using ELISA. An independent samples t-test and the Mann-Whitney U test were performed to compare variables between the two groups; one-way ANOVA and Kruskal-Wallis test were performed to compare four subgroups of patients. Correlations were determined by Spearman’s correlation tests. Stepwise multiple linear regression analyses were performed to assess for independent contributors. A receiver operating characteristic curve with the maximum Youden index was calculated for the optimal cut-off value.

**Results:**

In non-obese PCOS women, circulating OPN levels were increased in the subgroups with a higher body mass index (BMI) and free androgen index (FAI), and the LFC levels were increased in the elevated OPN subgroups. Moreover, increased OPN was associated with increased FAI and LFC in PCOS women, and the association between OPN and LFC was independent of triglyceride, HOMA-IR and FAI after adjusting for PCOS status in all participants. OPN combined with FAI and hsCRP may better predict NAFLD than WHR in this study cohort. However, there was no significant difference in circulating OPN levels between non-obese PCOS and normal control women.

**Conclusions:**

Increased OPN levels may be related to FAI and elevated LFC in non-obese women with PCOS.

**Electronic supplementary material:**

The online version of this article (10.1186/s12958-018-0331-4) contains supplementary material, which is available to authorized users.

## Background

Polycystic ovary syndrome (PCOS) is a complex reproductive and metabolic disorder related to insulin resistance with a 5–10% morbidity in women of fertile age [1]. As PCOS is characterized by insulin resistance and hyperandrogenism, it is often accompanied by metabolic syndrome and an increased risk of cardiovascular events [2]. It is believed that the occurrence and progression of metabolic disorders in PCOS patients are closely related to the chronic low-level inflammation of intra-abdominal adipose tissue [3, 4]. Among the metabolic disorders in PCOS patients, nonalcoholic fatty liver disease (NAFLD), which is considered the hepatic expression of metabolic syndrome [5], leads to a significant increase in the morbidity and pathology scores in PCOS patients, independent of obesity and other coexisting metabolic disorders [6–9].

NAFLD is an important manifestation of metabolic syndrome in the liver characterized by the presence of hepatic steatosis on imaging or histology in the absence of secondary causes of hepatic fat accumulation such as significant alcohol consumption, use of steatogenic medication or hereditary disorders [10]. A liver biopsy is the gold standard for the detection of liver fat content (LFC) in humans, but this invasive test is not routinely used in clinical practice; proton magnetic resonance ([1H]-MRS) [11, 12] and chemical shift-encoded magnetic resonance imaging (MRI) [13] are also accurate methods for assessing hepatic fat deposition, but they are not routinely performed due to high cost. A quantitative ultrasound method was recently established for detecting LFC and is proven to be a noninvasive method that provides results that are highly consistent with [1H]-MRS and histological liver steatosis grade (r = 0.85 and 0.79, respectively) [14]. Studies have shown that computer-aided measurements of the US hepatic/renal echo-intensity ratio (H/R) are highly correlated with liver fat content determined by histology and [1H]-MRS [15, 16]. The method used in this study was established based on a standardized H/R and the ultrasound hepatic echo-intensity attenuation rate (HA), which are reported to be positively correlated with the LFC measured by [1H]-MRS (r = 0.884, and 0.711, respectively) [17]. The ultrasound H/R can independently predict 78% of LFC detected by [1H]-MRS, and the addition of ultrasound HA improves the adjusted explained variance to 79.8% [17]. Compared to liver biopsy, MRS and MRI, the quantitative ultrasound method is non-invasive, time-saving, and relatively cheap; therefore, we chose to use the quantitative ultrasound method to assess the levels of LFC in this study population.

Osteopontin (OPN) is a glycoprotein that is involved in multiple biological and pathological conditions including immunity, inflammation, insulin resistance, reproduction, and steatosis and fibrosis of various tissues. OPN plays a role in immunomodulatory functions as an early T lymphocyte activator [18]. Studies demonstrate a significant increase of OPN levels in chronic low-grade inflammation and insulin resistance-related diseases such as obesity. Furthermore, OPN can induce inflammatory signalling from human adipocytes and adipose tissue macrophage infiltration, and impair differentiation and insulin sensitivity of primary adipocytes [19–22]. Growing evidence suggests that OPN is also involved in the regulation of female reproductive functions, such as follicular growth and ovulation regulation [23]. Recent studies have also found that plasma OPN levels are closely related to the degree of liver fibrosis in alcoholic liver disease [24]. Furthermore, previous studies suggest that OPN deficiency can reduce hepatic steatosis and inflammation in obese mice fed a high fat diet [21, 25]. In these studies, OPN expression was found to be essential for NAFLD progression, involving hepatic steatosis, inflammation and fibrosis. Therefore, it is now recognized that OPN is closely related to metabolic and reproductive disorders, as well as NAFLD. However, research on the correlation between serum OPN levels and LFC has not been evaluated in non-obese PCOS patients in the Chinese population.

The aim of this study was to assess whether OPN levels in non-obese PCOS patients are higher than that of age- and BMI-matched non-PCOS patients and the relationship between OPN levels and LFC.

## Methods

### Participants

Sixty-one women with PCOS and fifty-six control women were enrolled in the study from Shanghai, China. The age of the subjects ranged from 18 to 45 years old, and they were normal weight or overweight with a BMI < 28 kg/m^2^ according to the Cooperative Meta-Analysis Group of the Working Group on Obesity in China Criteria [26]; normal weight was defined as body mass index (BMI) < 24 kg/m^2^, overweight was defined as 24 kg/m^2^ ≤ BMI < 28 kg/m^2^, and obesity was defined BMI of ≥28 kg/m^2^. The diagnosis of PCOS was based on the National Institutes of Health 1990 Criteria [27]. Control women were screened by medical history, physical examination, laboratory evaluation and transvaginal ultrasound to select those with normal cycles, those who are non-hirsute, and those who do not have hyperandrogenaemia (serum testosterone below 0.6 ng/mL). All women that were pregnant (diagnosed by a urine pregnancy test) or had abnormal thyroid function and prolactin levels were excluded [27]. Women who received glucocorticoids, anti-androgen agents, oral contraceptives, anti-inflammatory agents, ovulation induction agents, anti-obesity medications, steatogenic medications, or insulin sensitizing agents within the previous 3 months were excluded. All women with history of significant alcohol consumption and hereditary disorders that might cause secondary hepatic fat accumulation were excluded.

All evaluations and procedures in this study were conducted according to the Declaration of Helsinki for Medical Research involving Human Subjects. This study was approved by the ethics committee of Shanghai Renji Hospital and written consent was obtained from each subject after full explanation of the purpose and nature of all study procedures.

### Quantitative measurement of LFC by ultrasonography

Quantitative measurement of LFC was determined by ultrasonography described in a previously study [14, 17]. All instrument settings were calibrated using a tissue-mimicking phantom (Model 057; Computerized Imaging Reference Systems, Norfolk, VA) and fixed before measurements were obtained to maintain consistency among the ultrasound machines. The phantom contains abdominal organ models that display as ultrasound images. The ultrasonographists were trained to obtain ultrasound images, including one image with both the liver and the right kidney clearly visualized in the sagittal liver/kidney view in the lateral position and another image with the liver in the right intercostal view at the anterior axillary line in the supine position [14, 17].

All images were transferred to a personal computer and analysed using the NIH image software (ImageJ 1.41, National Institutes of Health, Bethesda, MD). In the sagittal liver/right kidney view, a region of interest (ROI) of 1.5 * 1.5 cm^2^ in the liver parenchyma without blood vessels, bile ducts, and other focal hypo/hyperechogenic structures was selected. Another ROI of 0.5 * 0.5 cm^2^ was identified in the right renal cortex with no large vessels or renal sinus or medulla at the same depth as the liver ROI. In the right intercostal view at the anterior axillary line, two ROIs of 1.5 * 1.5 cm^2^ were selected in homogeneous regions of the liver along the same ultrasound transmission line near the liver’s anterior and posterior margins. The linear distance between the two ROIs was also measured. The grey scale mean value of the pixels within the ROIs was used as the measurement of echo intensity. We then divided the average hepatic grey scale by the average renal cortex grey scale to obtain the ultrasound hepatic/renal echo-intensity ratio. We obtained the standardized ultrasound hepatic/renal ratio and standardized hepatic attenuation rate using the phantom. The LFC was obtained according to the following predictive formula: LFC (%) = 62.592 * standardized ultrasound hepatic/renal ratio + 168.076* standardized hepatic attenuation rate – 27.863 [14, 17].

### Anthropometric measurements

The height and weight of each subject in light clothing were measured to the nearest 1 cm and 0.1 kg, respectively, using a digital scale and stadiometer. BMI was calculated as body weight (kg) divided by height (m) squared. Waist circumference (WC) and hip circumference (HC) were measured by a single individual. WC was determined by measuring the circumference at the narrowest point between the lower border of the rib cage and the iliac crest. HC was determined by measuring the circumference at the level of the symphysis pubis and the greatest gluteal protuberance. The waist-to-hip ratio (WHR) was then calculated by dividing the WC by the HC. Body fat percentage (FAT%) was assessed by foot-to-foot measures of bioelectrical impedance obtained using a TBF-300 body composition analyser (TANITA, U.K. Ltd., Middlesex, UK).

### Laboratory analyses

All laboratory evaluations were performed at 0800 h after an overnight fast during the early follicular phase (days 2–5) of a spontaneous menstrual cycle, except in subjects with amenorrhoea > 3 months who were examined randomly. All women underwent a standard oral glucose tolerance test (OGTT) with 75 g of glucose. Blood samples were drawn before the glucose load (t = 0 min) and after the glucose load (t = 30, 60, 120, and 180 min). Glucose and insulin samples were stored at 4 °C and analysed the day of sampling. All serum samples for OPN were stored at − 70 °C until assayed.

Competitive electrochemiluminescence immunoassays on the Elecsys Autoanalyser 2010 (Roche Diagnostics, Indianapolis, IN) were used to quantify serum total testosterone. Sex hormone binding globulin (SHBG) levels were measured by the chemiluminescent immunoassay (Elecsys autoanalyser 2010, Roche Diagnostics) validated for plasma SHBG. The coefficient of variation (CV) for SHBG using this methodology was 6%. FAI was calculated as the percentage ratio of total testosterone to SHBG [28]. A normal androgen level was defined as FAI < 7 [29]. Plasma glucose was determined using the glucose oxidase methodology. All measurements were performed with Roche reagents (D 2400 and E 170 Modular Analytics modules with Roche/Hitachi analysers; Roche Diagnostics, Indianapolis, USA). Insulin levels were measured by radioimmunoassay (RIA). The intra-assay CV of insulin and steroid hormone assays were 5.5% and < 10%, respectively. To estimate insulin resistance, the homeostasis assessment insulin resistance index (HOMA-IR) was calculated according to the equation fasting serum insulin (μU/ml) * fasting plasma glucose (mmol/ l)/22.5 [30]. The Matsuda Index was calculated using the eqs. 10,000/square root of [(fasting glucose x fasting insulin) x (mean glucose x mean insulin during OGTT)] [31]. The deposition index (DI) was calculated to estimate the β-cell response relative to the prevailing insulin sensitivity using the eq. DI = ΔI_30_/ΔG_30_ (mIU/mmol)/HOMA-IR = (I_30_-I_0_)/(G_30_-G_0_)/ HOMA-IR [32]. Analysis of high-sensitivity C-reactive protein (hsCRP) was performed using immunonephelometric methods and a BN-II analyser (Dade Behring, Deerfeld, Germany). The inter- and intra-assay coefficients of variation were 4.9% and 6.8%, respectively.

The plasma OPN level was measured using an enzyme-linked immunosorbent assay (ELISA) kit (R&D Systems, Minneapolis, MN, USA) according to the manufacturer’s instructions. The intra-assay CV was < 4.1% and the inter-assay CV was < 6.7%. All samples were analysed in duplicate. The assays have < 0.5% cross-reactivity observed with available related molecules and a < 50% cross-species reactivity observed with species tested.

### Statistical analyses

All statistical analyses were performed using SPSS version 23 (SPSS Inc., Chicago, IL, USA). The results are reported as the mean with the standard deviation for variables with a normal distribution and median with the interquartile range (25–75%) for variables with a skewed distribution. For variables with a normal distribution, an independent samples t-test was performed to compare variables between two groups; one-way ANOVA followed by the LSD test was performed for the four subgroups. For variables with a skewed distribution, the Mann-Whitney U test was performed to compare variables between the two groups; the Kruskal-Wallis test followed by the Mann-Whitney U test was performed for the four subgroups. The relationships between levels of OPN, LFC and other variables were evaluated by the Spearman’s correlation test. Stepwise multiple linear regression analyses were performed to assess the independent contributors of OPN and LFC. To determine the optimal threshold to predict LFC, the point on the receiver operating characteristic (ROC) curve with the maximum Youden index [sensitivity-(1-specificity)] was calculated. *P* < 0.05 was considered statistically significant.

## Results

### Clinical, hormonal and metabolic features of PCOS and non-PCOS women

The clinical characteristics and biochemical variables of PCOS and non-PCOS women are summarized in Table 1. The age, BMI, FAT%, HOMA-IR, DI, TG, and high-density lipoprotein cholesterol (HDL-C) and low-density lipoprotein cholesterol (LDL-C) levels were comparable between the PCOS and non-PCOS groups (all *P* > 0.05). However, compared to the non-PCOS group, the PCOS group had much higher WHR, AUCglucose, AUCinsulin, TC, hsCRP, T, FAI and A_2_, while the Matsuda Index and SHBG were lower in the PCOS group (all *P* < 0.05).

**Table 1:** Clinical characteristics and biochemical variables in women with and without PCOS

Parameters	PCOS			non-PCOS			*P* value
	Total	Lean	Overweight	Total	Lean	Overweight	(PCOS vs non-PCOS)
N	61	30	31	56	38	18	
Age, yrs	26(21–31)	24.5(21–31.25)^d^	26(23–31)	26(24–29)	25.5(24–27)	29.5(26.75–34)^a^	0.415
BMI, kg/m^2^	24.98(21.7–26.98)	21.7(19.41–23.3)^d^	26.89(25.63–27.77)^c,f^	22.06(21–24.91)	21.59(20.41–22.11)	27.48(24.91–27.85)^a^	0.062
WHR	0.87 ± 0.07	0.83 ± 0.07^d^	0.91 ± 0.06 ^c,f^	0.85 ± 0.07	0.82 ± 0.06	0.9 ± 0.07^a^	0.043
FAT%	32.5(28.5–37.3)	28.8(22.85–31.6)^d^	37.55(35.88–41.55)^c,f^	31.2(23.7–38.6)	24.25(21.3–27.3)	38.6(36.5–41.4)^a^	0.772
AUCglucose	10.69(9.38–12.54)	9.77(7.89–11.79)	11.63(10.49–13.06)^c,f^	9.69(8.1–11.66)	8.79(7.51–10.74)	10.73(9.73–12.6)^a^	0.018
AUCinsulin	107.57(54.18–161.07)	81.25(48.38–130.12)	137.51(91.62–193.94)^c,f^	73.46(45.57–114.58)	68.93(44.48–98.54)	83.72(43.96–137.69)	0.017
HOMA-IR	2.21(1.39–4.2)	1.62(0.92–2.31)^d^	3.47(2.14–4.96)^c^	1.93(1.19–2.69)	1.61(1–2.25)	3.22(1.85–4.46)^a^	0.066
Matsuda Index	65.61(40.35–105.8)	101.18(69.86–152.44)	49.8(37.46–63.53)^c,f^	100.13(63.46–154.98)	110.19(86.21–166.93)	70.21(42.8–113.74)^a^	0.008
DI	8.67(4.55–16.1)	14.17(7.23–20.69)^d^	6.14(2.37–9.79)^c,f^	10.94(5.48–22.47)	18.09(8.81–23.94)	5.49(1.98–7.43)^a^	0.213
TG, mmol/L	1.17(0.8–1.53)	0.87(0.72–1.18)^d^	1.26(1.09–1.78)^f^	1.29(0.77–1.6)	1.07(0.66–1.6)	1.48(1.15–1.93)^a^	0.704
TC, mmol/L	4.42(4.01–4.88)	4.14(3.91–4.75)^b,d^	4.6(4.01–5.18)^c^	4.2(3.41–4.89)	3.56(3.34–4.6)	4.66(4.34–5.16)^a^	0.024
HDL-C, mmol/L	1.25(1.08–1.54)	1.45(1.27–1.67)^d^	1.18(1.02–1.25)^c,f^	1.44(1.21–1.57)	1.47(1.34–1.65)	1.18(1.04–1.31)^a^	0.218
LDL-C, mmol/L	2.77 ± 0.83	2.49 ± 0.68^d^	3.04 ± 0.89^c,f^	2.55 ± 0.58	2.35 ± 0.53	2.96 ± 0.48^a^	0.101
hsCRP, mg/L	1.09(0.33–3.03)	0.54(0.22–1.35)^d^	2.04(0.55–4.15)^c,f^	0.37(0.17–1.65)	0.27(0.17–0.44)	1.82(0.74–3.91)^a^	0.014
T, nmol/	2.36(1.66–2.75)	2.36(1.4–2.83)^b,d^	2.4(1.83–2.63)^c,e^	1.63(1.39–1.87)	1.6(1.4–1.79)	1.73(1.02–2.03)	< 0.001
SHBG, nmol/L	23.3(14.6–45.7)	35.05(20.58–66.28)	18.7(11.3–29.7)^c,e,f^	41.75(30.73–60.05)	50.2(38.7–62.75)	32.85(18.58–39.6)^a^	< 0.001
FAI	9.29(4.46–15.25)	5.57(2.81–11.43)^b^	12.54(7.27–19.73)^c,e,f^	3.61(2.48–5.2)	3.19(2.45–4.44)	5.4(2.61–7.73)^a^	< 0.001
A_2_, μg/dL	5.9(4.22–7.98)	4.52(2.14–6.97)	6.49(4.83–9.5)^c,e^	2.85(2.24–5.52)	2.97(2.26–5.64)	2.73(2.11–5.83)	0.019

Data are shown as the mean ± SD for variables with a normal distribution and median with the interquartile range (25–75%) for skewed variables. For variables with a normal distribution, an independent samples t-test was performed to compare variables between two groups; a one-way ANOVA followed by the LSD test was performed among the four subgroups of women (lean control, overweight control, lean PCOS, and overweight PCOS). For variables with a skewed distribution, the Mann-Whitney U test was performed to compare variables between the two groups; the Kruskal-Wallis test followed by the Mann-Whitney U test was performed for the four subgroups of women

*BMI* Body mass index, *WC* Waist circumference, *WHR* Waist hip ratio, *FAT*% Body fat percentage, *AUCglucose* Area under curve for OGTT glucose, *AUCinsulin* Area under curve for OGTT insulin, *HOMA-IR* Homeostasis model assessment of insulin resistance, *DI* Disposition index, *TG* Triglyceride, *TC* Total cholesterol, *HDL-C* High-density lipoprotein cholesterol, *LDL-C* Low-density lipoprotein cholesterol, *hsCRP* high sensitivity C reactive protein, *T* Total testosterone, *SHBG* Sex hormone binding globulin, *FAI* Free androgen index *A*_2_ androstenedione

To convert testosterone to ng/ml, divide by 3.467; To convert cholesterol to mg/dL, divide by 0.02586; to convert triglycerides to mg/dL, divide by 0.01129; to covert glucose to mg/dL, divide by 0.05551; to convert total testosterone to ng/ml, divide by 3.467

^a^*P* < 0.05 for lean non-PCOS women vs overweight non-PCOS women

^b^*P* < 0.05 for lean non-PCOS women vs lean PCOS women

^c^*P* < 0.05 for lean non-PCOS women vs overweight PCOS women

^d^*P* < 0.05 for overweight non-PCOS women vs lean PCOS women

^e^*P* < 0.05 for overweight non-PCOS women vs overweight PCOS women

^f^ P < 0.05 for lean PCOS women vs overweight PCOS women

The PCOS and non-PCOS women were divided into four subgroups according to their BMI. Lean PCOS and lean non-PCOS women did not differ except in levels of TC and sex hormones (T and FAI) (*P* < 0.05 for all). When we compared overweight non-PCOS and overweight PCOS women, there were similarities in metabolic parameters, while their sex hormones including T, SHBG, A_2_ and FAI, were quite different (*P* < 0.05 for all). Other clinical, metabolic and hormonal parameters are shown in Additional file 1: Table S1.

### Comparison of OPN levels in different subgroups

**Fig. 1:**
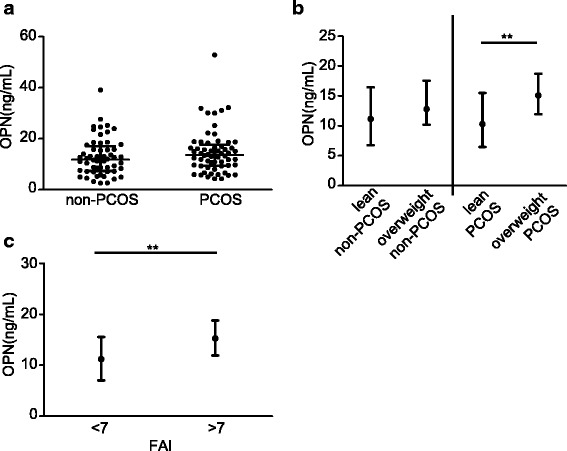
OPN levels in the lean/overweight non-PCOS and PCOS groups and in FAI quartile groups. (**a**) OPN levels in the non-PCOS group and PCOS group. (**b**) OPN levels in lean and overweight non-PCOS groups and lean and overweight PCOS groups. (**c**) OPN levels by FAI in the PCOS group. For comparison between two groups, the Mann-Whitney U test was performed. The Kruskal-Wallis test followed by the Mann-Whitney U test was performed to test the differences among the four subgroups. Data are displayed as median with interquartile range. *P < 0.05, **P < 0.01. FAI: free androgen index.

The mean value of OPN was moderately higher in PCOS patients compared to non-PCOS women, but this finding was not statistically significant (13.65 ng/mL vs 11.78 ng/mL, *P* = 0.160, Fig. 1a). Furthermore, we divided all participants into four subgroups according to PCOS and BMI status and compared the mean values of OPN. We observed that the mean value of OPN in overweight PCOS patients was significantly higher than that of lean PCOS patients (15.09 vs 10.31 ng/mL, *P* = 0.01, Fig. 1b), while there was no difference between lean control women and overweight control women (11.15 vs 12.81 ng/mL, *P* = 0.229, Fig. 1b). Similar levels of OPN were also found between lean control women and lean PCOS patients and between overweight control women and overweight PCOS women (both *P* > 0.05, Fig. 1b). Since hyperandrogenism is an important characteristic of PCOS patients, we explored whether androgen has an impact on the OPN level. We compared OPN levels and FAI to identify whether OPN levels change with bioactive testosterone estimated by FAI. In PCOS patients, the OPN levels were higher in those with FAI > 7 compared to those with FAI < 7 (9.61 ng/mL vs 15.22 ng/mL, *P* = 0.007, Fig. 1c). The baseline data according to FAI in PCOS women are shown in Additional file 1: Table S2.

### Comparison of LFC levels in different subgroups by PCOS status, BMI and OPN

**Fig. 2:**
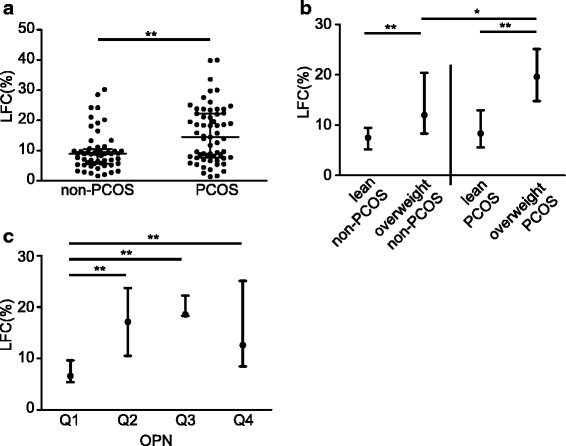
LFC levels in lean/overweight non-PCOS and PCOS groups and in OPN quartile groups. (**a**) LFC levels in non-PCOS and PCOS groups. (**b**) LFC levels in lean and overweight non-PCOS groups and lean and overweight PCOS groups. (**c**) LFC levels by OPN quartiles in the PCOS group For comparison between PCOS and non-PCOS groups, the Mann-Whitney U test was performed. The Kruskal-Wallis test followed by the Mann-Whitney U test was performed to test the differences among the four subgroups. Data are displayed as median with interquartile range. *P < 0.05, **P < 0.01. LFC: liver fat content.

The mean value of LFC was significantly higher in PCOS patients compared to age- and BMI- matched non-PCOS women (14.47% vs 8.93%, *P* = 0.002, Fig. 2a). All participants were then divided into different subgroups according to BMI and diagnosis. In the PCOS group, the mean value of LFC in overweight patients was significantly higher than that of lean patients (19.61% vs 8.32%, *P* < 0.001, Fig. 2b). In the non-PCOS group, the mean value of LFC in overweight women was significantly higher than that of lean women (11.95% vs 7.47%, *P* = 0.001, Fig. 2b). The mean value of LFC was significantly higher in overweight PCOS patients compared to that of overweight non-PCOS patients (19.61% vs 11.95%, *P* = 0.021, Fig. 2b). However, lean PCOS patients and lean non-PCOS women were observed to have similar LFC levels (*P* = 0.348, Fig. 2b).

To explore whether OPN affects LFC in PCOS women, we further divided the subjects into subgroups according to the quartiles of OPN levels and compared LFC levels among these four subgroups. In PCOS subjects, LFC was higher in women with OPN levels in the second (6.6% vs 17.14% *P* = 0.002), third (6.6% vs 18.58% *P* < 0.001) and fourth quartiles (6.6% vs 12.59% *P* = 0.004, Fig. 2c) compared to those with OPN levels in the lowest quartile. The baseline data of the four subgroups according to OPN levels in PCOS women are shown in Additional file 1: Table S3.

### Correlation between OPN and LFC with clinical/biochemical parameters in PCOS patients

Given the association between high OPN levels and increased adiposity, correlations between OPN and anthropometric/metabolic parameters were investigated (Table 2). In PCOS women, the OPN level had a positive linear relationships with LFC (*P* = 0.004). Moreover, OPN level positively correlated with AUCinsulin (*P* = 0.016), HOMA-IR (*P* = 0.02), and FAI (*P* = 0.016). Furthermore, there were strong inverse linear correlations between OPN level and SHBG (*P* = 0.027) and the Matsuda Index (*P* = 0.037). However, we did not find a correlation between OPN level and anthropometry parameters, lipid profile, hsCRP, AUCglucose, or DI (Table 2).

**Table 2:** Correlation of OPN and LFC with clinical and biochemical parameters in PCOS women

	OPN		LFC	
	*r*	*P*	*r*	*P*
OPN	/	/	0.36	0.004
liver	0.360	0.004	/	/
BMI	0.246	0.056	0.621	< 0.001
WHR	0.118	0.372	0.514	< 0.001
FAT%	0.239	0.273	0.569	0.005
AUCglucose	0.203	0.116	0.495	< 0.001
AUCinsulin	0.321	0.016	0.488	< 0.001
HOMA-IR	0.300	0.02	0.474	< 0.001
Matusda index	−0.282	0.037	− 0.569	< 0.001
DI	−0.082	0.546	−0.337	0.01
TG	0.067	0.607	0.464	< 0.001
HDL-C	−0.208	0.107	−0.514	< 0.001
LDL-C	−0.093	0.476	0.263	0.041
hsCRP	0.137	0.291	0.375	0.003
SHBG	−0.283	0.027	−0.533	< 0.001
FAI	0.308	0.016	0.443	< 0.001

Associations between OPN and LFC with other variables were determined by the Spearman correlation analysis

*LFC* Liver fat content, *BMI* Body mass index, *WHR* Waist-to-hip ratio, *FAT*% Body fat percentage, *AUCglucose* Area under curve for OGTT glucose, *AUCinsulin* Area under curve for OGTT insulin, *HOMA-IR* Homeostasis model assessment of insulin resistance, *DI*: disposition index, *TG* Triglyceride, *HDL-C* High-density lipoprotein cholesterol, *LDL-C* Low-density lipoprotein cholesterol, *hsCRP* High sensitive C reaction protein, *SHBG* Sex hormone binding globulin, *FAI* Free androgen index

Given the association between high LFC levels and increased adiposity, correlations between LFC and anthropometric/metabolic parameters were investigated (Table 2). In PCOS women, the LFC level had positive linear relationships with BMI, FAT% and WHR (*P* < 0.01 for all). LFC level also had positive linear relationships with AUCglucose (*P* < 0.001), AUCinsulin (*P* < 0.001), HOMA-IR (*P* < 0.001), TG (*P* < 0.001), LDL-C (*P* = 0.041), hsCRP (*P* = 0.003), and FAI (*P* < 0.001). Furthermore, strong inverse linear correlations were found between LFC level and the Matsuda Index (*P* < 0.001), DI (*P* = 0.01), SHBG (*P* < 0.001) and HDL-C (*P* < 0.001, Table 2).

### Determinants of LFC

To investigate the independent determinants of LFC among all women studied, we performed a stepwise multiple regression analysis on LFC with OPN, HOMA-IR, FAI, TG and PCOS status as potential contributors. We observed that FAI, HOMA-IR, TG and OPN, but not PCOS status, explained 33.5% of the variance in LFC (adjusted R^2^ = 0.335, *P* < 0.001) with FAI (*β* = 0.347, *P* < 0.001), HOMA-IR (*β* = 0.201, *P* = 0.013), TG (*β* = 0.236, *P* = 0.004) and OPN (*β* = 0.178, *P* = 0.023, Table 3) being significant independent contributors. Therefore, FAI, HOMA-IR, TG and OPN were all found to be independent predictors of LFC.

**Table 3:** Effects of OPN and clinical parameters on LFC in all participants adjusted for PCOS status

		95%CI		*P* value
	*β*	Lower Bound	Upper Bound	
FAI	0.326	0.178	0.473	< 0.001
TG	3.199	1.068	5.33	0.004
HOMA-IR	0.671	0.146	1.197	0.013
OPN	0.189	0.026	0.352	0.023

Stepwise multiple linear regression analyses were performed to evaluate the effects of FAI, TG, HOMA-IR and OPN on LFC adjusted for PCOS status

*LFC* Liver fat content, *FAI* Free androgen index, *TG* Triglyceride, *HOMA-IR* Homeostasis model assessment of insulin resistance

### ROC analysis for LFC

To further elucidate the relationship between OPN and LFC, we used the ROC curve to find potential contributors of elevated LFC risk for NAFLD diagnosis (LFC > 5%). In all participants, the area under the curve (AUC) in the ROC analysis was 0.606 for OPN, 0.615 for FAI and 0.691 for hsCRP. We combined the three potential contributors of LFC and found that the AUC increased to 0.71 from 0.622 for WHR, indicating that the combination of OPN, FAI and hsCRP are optimal predictors for the diagnosis for NAFLD (Table 4).

**Table 4:** ROC curve analysis of LFC risk (> 5%) in all participants

	Cut-off point	AUC	Sensitivity	Specificity
WHR	0.905	0.622	0.35	1
OPN	13.005	0.606	0.51	0.8
hsCRP	0.77	0.691	0.51	0.867
FAI	6.155	0.615	0.441	0.8
OPN + FAI + hsCRP		0.71	0.667	0.8

WHR Waist-hip ratio, *hsCRP* High sensitive C reaction protein, *FAI* Free androgen index

## Discussion

In this study, we investigated circulating OPN levels in non-obese women with PCOS compared to normal control women and determined the correlation between OPN and LFC. The main finding of this study was that the circulating OPN level is associated with the degree of FAI in non-obese PCOS women and the OPN is an independent contributor of LFC. OPN combined with FAI and hsCRP was an optimal predictor of NAFLD in this study cohort. However, there was no significant difference in circulating OPN levels between non-obese PCOS and normal control women.

There is an increasing number of studies investigating the relationship between OPN and metabolic diseases, but there have been no definitive conclusions to date. Whether OPN independently contributes to the development of metabolic disturbance in PCOS patients is also unknown. Because OPN has multiple functions in adipose tissue, we hypothesized that OPN may be involved in the insulin resistance in PCOS patients. Although differences in insulin resistance estimated by HOMA-IR were not readily apparent between PCOS and non-PCOS women in this non-obese cohort, the level of SHBG, a potential alternative index of insulin resistance, was significantly decreased in non-obese women with PCOS. Our study also suggests that OPN levels are negatively correlated with SHBG levels.

Since hyperandrogenism is one of the most important features of PCOS pathogenesis, we analysed the differences in OPN levels in different FAI subgroups of PCOS patients. We found that an increased circulating OPN level was more common in higher FAI groups and associated with high degree of FAI in non-obese PCOS patients. This finding is consistent with a recent study that showed that OPN levels are associated with free testosterone levels [33]. In the BMI subgroup analysis, OPN levels were significantly higher in the overweight PCOS group compared to the lean PCOS group, while this difference was not found in control women. This suggests that hyperandrogenism may play an important role in elevating OPN levels, for there is only a small BMI difference among patients with hyperandrogenaemia.

In the present study, there was no significant difference in serum OPN levels between non-obese PCOS and control women. This finding is inconsistent with that of a recent study showing that PCOS is associated with increased serum OPN levels [33]. The major discrepancy may be due to the relatively low BMI and comparable HOMA-IR in our cohort. However, given the observational design of our study and Saklamaz’s study, causality cannot be established. The precise mechanisms of the potential role of OPN in PCOS must be further investigated.

While there is growing evidence with regard to OPN playing a crucial role in metabolic disorders, particularly focusing on NAFLD and obesity, data on OPN and LFC in PCOS are rather limited. To our knowledge, this is the first report describing the increased circulating OPN levels associated with a high degree of LFC in non-obese PCOS women. In line with a previous study, despite differences in BMI of the patient cohorts, OPN was observed to be correlated with several metabolic parameters such as BMI, WHR (waist circumference in a previous study), glucose and insulin levels, HOMA-IR, TG, HDL-C, LDL-C, and hsCRP [34]. Moreover, PCOS patients with OPN levels in the third quartile showed a 2.82 times greater LFC than those in the PCOS group with OPN levels in the lowest quartile (18.58% vs 6.6%). Interestingly, compared to those in the second and third quartiles, subjects with OPN levels in the highest quartile had a lower LFC. Since OPN is elevated in the progression of simple steatosis to nonalcoholic steatohepatitis and fibrosis [34], the change in LFC levels may account for this decline in OPN. In this study, we excluded fibrosis using the BMI, age, ALT and triglycerides (BAAT) index because of the relatively low BMI and age in this cohort. In addition, a stepwise linear regression analysis showed that the serum OPN level is a predictor of LFC independent of TG, HOMA-IR and FAI, indicating that the effect of changes in OPN levels on the metabolic phenotype of PCOS may be the result of a mechanism that is independent of insulin sensitivity.

There are several limitations in this study. First, the observational design cannot prove causality. Second, we quantitatively determined the LFC levels by ultrasonography. Although [1H]-MRS is the most accurate, non-invasive and quantitative LFC detection method based on a large-scale population study, there is sufficient correlation in LFC detection between quantitative ultrasonography and MRS. In addition, we detected serum OPN levels by ELISA, which has been found to be consistent with the results of western blot analyses [35].

## Conclusions

In conclusion, the levels of circulating OPN in non-obese Chinese women positively correlated with the FAI and LFC levels. Furthermore, OPN is an independent predictor of LFC in PCOS women and may contribute to the metabolic phenotypes of PCOS through mechanisms independent of insulin resistance. In addition, OPN combined with FAI and hsCRP may be an optimal predictor for NAFLD in PCOS. Further studies are needed to elucidate the mechanism of OPN in the development of NAFLD in PCOS patients.

## Additional file

Additional file 1: Supplementary Tables. (DOCX 21 kb)
