# Supplementary material for: Circulating osteopontin and its association with liver fat content in non-obese women with polycystic ovary syndrome: a case control study
Source: Reprod Biol Endocrinol. 2018 Mar 27;16:31. doi: 10.1186/s12958-018-0331-4 (PMC5870073; doi:10.1186/s12958-018-0331-4)
Supplement: Supplementary file 1 — Supplementary Tables. (DOCX 21 kb) [file 12958_2018_331_MOESM1_ESM.docx]

**Supplementary Table 1: Supplementary clinical characteristics and biochemical variables in women with PCOS and non-PCOS.**

| **Parameters** | **PCOS** | | | **non-PCOS** | | | ***P value*** |
| --- | --- | --- | --- | --- | --- | --- | --- |
|  | **Total** | **Lean** | **Overweight** | **Total** | **Lean** | **Overweight** | **(PCOS *vs***  **non-PCOS)** |
| **N** | 61 | 30 | 31 | 56 | 38 | 18 |  |
| **WC, cm** | 86.29+11.78 | 77.09+7.86^d^ | 94.6+7.9^cf^ | 81.81+10.78 | 76.26+4.81 | 93.53+10.56^a^ | 0.036 |
| **FBG, mmol/L** | 4.86(4.53-5.21) | 4.85(4.42-5.07)^d^ | 4.88(4.54-5.31) | 4.87(4.7-5.17) | 4.79(4.52-5.04) | 5.09(4.9-5.39)^a^ | 0.602 |
| **2hBG, mmol/L** | 7.07(5.86-8.28) | 5.94(5.39-7.36) | 7.66(6.66-9.78)^cef^ | 6.03(5.05-7.18) | 5.59(5.02-6.65) | 6.66(6.05-7.92)^a^ | 0.002 |
| **FINS, mIU/L** | 10.67(6.45-18.44) | 7.31(4.65-11.29)^d^ | 17.69(9.4-21.64)^cf^ | 8.51(5.66-12.08) | 7.72(4.74-10.58) | 14.53(7.6-18.61) ^a^ | 0.051 |
| **2hINS, mIU/L** | 71.18(38.03-127.57) | 48.61(33.17-95.36) | 102.26(54.49-139.97)^cef^ | 51.46(28.38-75.89) | 44.77(26.14-67.69) | 60.07(43.08-93.49) | 0.017 |
| **LH, IU/L** | 8.88(4.39-15.61) | 8.06(4.17-18.95)^bd^ | 9.03(4.4-12.58)^c^ | 4.99(2.98-9.14) | 4.99(3.07-8.46) | 4.93(2.78-11.77) | 0.004 |
| **FSH, IU/L** | 6.92(5.46-9.61) | 8.43(5.9-10.4) | 6.47(4.8-9.3) | 7.78(6.84-9.17) | 7.69(6.84-9.01) | 8.18(6.83-9.42) | 0.204 |
| **DHEA_S_,ng/mL** | 227.61+96.87 | 228.29+101.7 | 227+94.06 | 203.74+78.61 | 200.99+71.58 | 209.74+94.49 | 0.161 |

Data are shown as mean±SD for variables of normal distribution, and median with the interquartile range (25-75%) for skewed variables. For varibles of normal distribution, independent sample t-test was performed to compare variables between two groups; one-way ANOVA followed by LSD test were performed considering four subgroups of women: lean control, overweight control, lean PCOS, and overweight PCOS. For variables of skewed distribution, Mann-Whitney U test was performed to compare variables between two groups; Kruskal-Wallis test followed by Mann-Whitney U test were performed considering four subgroups of women.

WC: waist circumference; FBG: fasting blood glucose; 2hBG: 2-hour glucose; FINS: fasting insulin; 2hINS: 2-hour insulin; LH: luteinizing hormone; FSH: follicle-stimulating hormone; DHEA_S_: dehydroepiandrosteronesulfate.

To covert glucose to mg/dL:, divide by 0.05551.

a: P<0.05 for lean non-PCOS women *vs* overweight non-PCOS women.

b: P<0.05 for lean non-PCOS women *vs* lean PCOS women.

c: P<0.05 for lean non-PCOS women *vs* overweight PCOS women.

d: P<0.05 for overweight non-PCOS women *vs* lean PCOS women.

e: P<0.05 for overweight non-PCOS women *vs* overweight PCOS women.

f: P<0.05 for lean PCOS women *vs* overweight PCOS women.

**Supplementary Table 2: Clinical characteristics and biochemical variables of different FAI in PCOS women.**

|  | **PCOS** | | ***P***  **value** |
| --- | --- | --- | --- |
|  | **FAI<7** | **FAI>7** |  |
| **N** | 22 | 39 |  |
| **Age, yrs** | 27.5(21.75-31.25) | 25(21-30) | 0.185 |
| **Weight, kg** | 58.95(52.35-69.1) | 68.2(61.6-75) | 0.015 |
| **BMI, kg/m2** | 22.37(19.51-25.13) | 25.63(23.28-27.65) | 0.01 |
| **WC, cm** | 79.25(72-93) | 89.5(82-94.75) | 0.038 |
| **WHR** | 0.85+0.08 | 0.89+0.07 | 0.04 |
| **FAT%** | 29.77+8.19 | 33.74+6.64 | 0.213 |
| **FBG, mmol/L** | 4.64(4.39-5.14) | 4.89(4.59-5.21) | 0.247 |
| **2hBG, mmol/L** | 6.4(5.48-7.57) | 7.31(5.95-8.36) | 0.118 |
| **FINS, mIU/L** | 7.94(4.65-15.14) | 12.02(7.83-19.67) | 0.083 |
| **2hINS, mIU/L** | 49.35(29.88-118.97) | 82.06(46.87-132.26) | 0.057 |
| **AUCglucose** | 10.16(8.05-11.88) | 11.01(9.75-12.64) | 0.093 |
| **AUCinsulin** | 86.3(44.41-150) | 122.87(72.46-180.9) | 0.177 |
| **HOMA-IR** | 1.78(0.92-3.29) | 2.83(1.67-4.3) | 0.053 |
| **Matsuda Index** | 99.71(63.53-150.14) | 56.29(37.67-104.42) | 0.025 |
| **DI** | 10.39(2.04-20.3) | 7.9(5.38-14.14) | 0.763 |
| **TG, mmol/L** | 0.88(0.71-1.27) | 1.18(0.84-1.78) | 0.07 |
| **TC, mmol/L** | 4.62(3.98-4.8) | 4.28(4.01-5.07) | 0.875 |
| **HDL-C, mmol/L** | 1.49(1.21-1.63) | 1.19(1.06-1.44) | 0.021 |
| **LDL-C, mmol/L** | 2.53+0.76 | 2.9+0.85 | 0.103 |
| **hsCRP, mg/L** | 0.48(0.17-1.7) | 1.69(0.48-3.8) | 0.027 |
| **LH, IU/L** | 9.18(3.62-18.68) | 8.63(5.31-12.58) | 0.486 |
| **FSH, IU/L** | 7.02(6.04-10.23) | 6.73(4.8-9.6) | 0.601 |
| **T, nmol/** | 1.67(1.14-2.46) | 2.51(2.05-2.79) | 0.005 |
| **SHBG, nmol/L** | 58.9(38.15-104.75) | 18.4(11.3-23.3) | <0.001 |
| **FAI** | 3.37(1.5-4.74) | 12.81(9.33-19.71) | <0.001 |
| **DHEA_S_, ng/mL** | 166.48+87.04 | 260.65+86.03 | <0.001 |
| **A_2_, ug/dL** | 3.31(1.96-8.49) | 6.18(4.51-8.49) | 0.301 |

Data are shown as mean±SD for variables of normal distribution, and median with the interquartile range (25-75%) for skewed variables. For varibles of normal distribution, independent sample t-test was performed to compare variables between two groups. For variables of skewed distribution, Mann-Whitney U test was performed to compare variables between two groups.

BMI: body mass index; WC: waist circumference; WHR: waist hip ratio; FAT%: body fat percentage; FBG: fasting blood glucose; 2hBG: 2-hour glucose; FINS: fasting insulin; 2hINS: 2-hour insulin; AUCglucose: area under curve for OGTT glucose; AUCinsulin: area under curve for OGTT insulin; HOMA-IR: homeostasis model assessment of insulin resistance; DI: disposition index; TG: triglyceride; TC: total cholesterol; HDL-C: high-density lipoprotein cholesterol; LDL-C: low-density lipoprotein cholesterol; hsCRP: high sensitivity C reactive protein; T: total testosterone; LH: luteinizing hormone; FSH: follicle-stimulating hormone; SHBG: sex hormone binding globulin; FAI: Free androgen index; DHEA_S_: dehydroepiandrosteronesulfate; A2: androstenedione.

To convert testosterone to ng/ml, divide by 3.467; To convert cholesterol to mg/dL, divide by 0.02586; to convert triglycerides to mg/dL, divide by 0.01129; to covert glucose to mg/dL:, divide by 0.05551; to convert total testosterone to ng/ml, divide by 3.467.

**Supplementary Table 3: Clinical characteristics and biochemical variables in PCOS women of different OPN quartiles..**

|  | | | **OPN** | | |
| --- | --- | --- | --- | --- | --- |
|  | **Q1** | **Q2** | | **Q3** | **Q4** |
| **N** | 15 | 16 | | 15 | 15 |
| **Age, yrs** | 28.47+4.02 | 25.38+4.47 | | 22.4+4.9^b^ | 28.53+7.95^d^ |
| **Weight, kg** | 57.61+9.89 | 67.36+8.57^a^ | | 69.01+12.22^b^ | 66.31+11.3^c^ |
| **BMI, kg/m2** | 22.34(19.43-23.37) | 25.26(22.51-27.65)^a^ | | 26.8(22.1-27.77)^b^ | 25.43(21.6-26.72)^c^ |
| **WC, cm** | 80.07+11.18 | 87.53+8.33 | | 90.04+12.28 | 87.79+13.77 |
| **WHR** | 0.86+0.08 | 0.87+0.06 | | 0.9+0.06 | 0.87+0.09 |
| **FAT%** | 29.5(23.55-37.4) | 31.6(28.8-32.6) | | 36.9(24.95-39.5) | 36.9(29.8-37.85) |
| **FBG, mmol/L** | 4.99(4.5-5.21) | 4.94(4.45-5.23) | | 4.86(4.59-4.91) | 4.68(4.42-5.37) |
| **2hBG, mmol/L** | 5.87(5.2-7.32) | 7.27(5.49-8.43) | | 7.69(6.65-8.84) | 7(6-8.36) |
| **FINS, mIU/L** | 6.9(4.68-9.5) | 12.17(7.71-19.73) | | 15.73(6.19-19.96) | 13.12(7.54-19.87) |
| **2hINS, mIU/L** | 37.69(24.83-59.11) | 97.58(46.55-140.6)^a^ | | 81.99(56.79-132.26)^b^ | 85.38(44.08-131.96)^c^ |
| **AUCglucose** | 10.25(8.54-11.01) | 10.64(9.31-14.19) | | 11.98(10.69-12.66) | 10.52(9.63-12.53) |
| **AUCinsulin** | 70.38+52.69 | 142.21+78.95^a^ | | 135.4+61.07^b^ | 121.51+65.69^c^ |
| **HOMA-IR** | 1.55(0.92-2.1) | 2.78(1.79-4.66) | | 3.42(1.35-4.33) | 2.89(1.61-4.29) |
| **Matsuda Index** | 104.11(83.48-139.82) | 55.91(42.39-96.24) | | 56.37(38.37-100.37) | 61.62(36.68-106.46) |
| **DI** | 10.49(2.07-18.26) | 8.67(4.37-10.28) | | 7.62(5.73-27.32) | 7.94(2.25-14.3) |
| **TG, mmol/L** | 0.87(0.77-1.17) | 1.23(0.94-1.8) | | 1.24(1.02-2.09)^b^ | 0.82(0.62-1.44)^d^ |
| **TC, mmol/L** | 4.8+0.97 | 4.54+0.95 | | 4.31+0.5 | 4.6+1.05 |
| **HDL-C, mmol/L** | 1.54(1.29-1.87) | 1.16(0.95-1.51)^a^ | | 1.12(0.99-1.25)^b^ | 1.38(1.19-1.5) |
| **LDL-C, mmol/L** | 2.76(2.13-3.72) | 2.96(2.3-3.24) | | 2.42(2.18-2.9) | 2.58(2.13-3.28) |
| **hsCRP, mg/L** | 0.5(0.29-1.96) | 1.66(0.23-3.11) | | 1.09(0.58-4.77) | 1.53(0.23-4.07) |
| **LH, IU/L** | 8.64+7.06 | 10.92+5.12 | | 14.93+15.07 | 9.89+8 |
| **FSH, IU/L** | 6.87+2.89 | 7.97+2.85 | | 7.75+4.83 | 8.02+2.95 |
| **T, nmol/** | 1.82+0.9 | 2.5+0.79 | | 2.24+0.6 | 2.57+1.07 |
| **SHBG, nmol/L** | 39.6(29.7-107) | 22.75(11.23-45.2)^a^ | | 18.4(11.6-21)^b^ | 28.2(19.3-34.7)^c^ |
| **FAI** | 3.01(1.31-8.62) | 12.25(5.37-26.09)^a^ | | 11.91(8.37-18.01)^b^ | 9.29(7.27-13.49)^c^ |
| **DHEA_S_, ng/mL** | 200.34+115.34 | 261.79+102.33 | | 258.39+89.11 | 187.72+56.88 |
| **A2, ug/dL** | 2.73(1.87-5.67) | 10(4.52-10) | | 5.9(4.56-7.48) | 6.52(4.22-10) |

Data are shown as mean±SD for variables of normal distribution, and median with the interquartile range (25-75%) for skewed variables. For varibles of normal distribution, one-way ANOVA followed by LSD test were performed considering four subgroups of women. For variables of skewed distribution, Kruskal-Wallis test followed by Mann-Whitney U test were performed considering four subgroups of women.

BMI: body mass index; WC: waist circumference; WHR: waist hip ratio; FAT%: body fat percentage; FBG: fasting blood glucose; 2hBG: 2-hour glucose; FINS: fasting insulin; 2hINS: 2-hour insulin; AUCglucose: area under curve for OGTT glucose; AUCinsulin: area under curve for OGTT insulin; HOMA-IR: homeostasis model assessment of insulin resistance; DI: disposition index; TG: triglyceride; TC: total cholesterol; HDL-C: high-density lipoprotein cholesterol; LDL-C: low-density lipoprotein cholesterol; hsCRP: high sensitivity C reactive protein; T: total testosterone; LH: luteinizing hormone; FSH: follicle-stimulating hormone; SHBG: sex hormone binding globulin; FAI: Free androgen index; DHEA_S_: dehydroepiandrosteronesulfate; A2: androstenedione.

To convert testosterone to ng/ml, divide by 3.467; To convert cholesterol to mg/dL, divide by 0.02586; to convert triglycerides to mg/dL, divide by 0.01129; to covert glucose to mg/dL:, divide by 0.05551; to convert total testosterone to ng/ml, divide by 3.467.

a: P<0.05 for Q1 *vs* Q2. b: P<0.05 for Q1 *vs* Q3. c: P<0.05 for Q1 *vs* Q4. d: P<0.05 for Q3 *vs* Q4.
